# Struvite precipitation from source-separated human urine: mineralogical characterization, phosphorus release kinetics, and heavy metal safety assessment

**DOI:** 10.1007/s11356-025-37380-6

**Published:** 2026-01-24

**Authors:** Ricardo Franci Gonçalves, Regiane Pereira Roque, Yuri Nascimento Nariyoshi, Renata Estevam, Honerio Coutinho de Jesus

**Affiliations:** Departamento de Engenharia Ambiental, Universidade Federal do Espirito Santo, Centro Tecnológico, Av Fernando Ferrari 514, Vitória, 29075-910 Brazil

**Keywords:** Phosphorus Recovery, Struvite Precipitation, Source-Separated Urine, Material Characterization, Sustainable Sanitation, Circular Economy

## Abstract

The recovery of phosphorus from human urine via struvite precipitation has emerged as a promising strategy for sustainable nutrient management. However, limited understanding of the materials' physicochemical properties, phosphorus speciation, and potential contamination by tracing heavy metals continues to hinder their safe application. This study addresses these gaps by systematically characterizing struvite precipitated from source-separated human urine collected from dry toilet facilities and stored at ambient temperature for six months, representing a scarcely explored effluent matrix in nutrient recovery research. The precipitation was driven by natural microbial urease activity and by the addition of magnesium oxide (MgO) without pH adjustment (Mg:P molar ratio of 1.71:2.21). The resulting solids were analyzed via X-ray diffraction (XRD), solid-state phosphorus-31 nuclear magnetic resonance ^31^P NMR, inductively coupled plasma atomic emission spectrometry (ICP-OES), and the Measurement and Standard Testing (MTP) protocol. Struvite (MgNH₄PO₄∙6H₂O) was identified as the dominant crystalline phase (79.54%), with minor presence of newberyite (MgHPO₄∙3H₂O) revealed by ^31^P NMR. Heavy metals, including arsenic, cadmium, lead, chromium, and mercury, were detected at concentrations well below regulatory thresholds established by Brazilian legislation. Phosphorus speciation analysis showed a predominance of inorganic forms (70.0 mg∙g⁻^1^ IP) within the product, which contained 10.2% P. Additionally, 61% of the total phosphorus content was solubilized within 10 days at neutral pH, with the kinetics excellently described by the Elovich model. These findings support the feasibility of converting human urine into a safe, phosphorus-rich fertilizer, advancing circular economy principles towards sustainable sanitation practices.

## Introduction

The recovery of nutrients from sanitation waste has emerged as a central strategy for valorizing underutilized resources while mitigating the environmental impacts of conventional wastewater management (Wang et al. 2023). Source-separated human urine is particularly relevant among these waste streams, accounting for over 70–80% of nitrogen and approximately 50–65% of phosphorus loads in domestic effluents (Saliu et al. 2024). This disproportionate contribution positions human urine as a strategic feedstock for nutrient circularity within sustainable sanitation frameworks.

Besides reducing nutrient losses and environmental impacts, nutrient recovery from sanitation waste fits into the long history of chemical innovations in plant nutrition. These innovations have gradually tackled resource shortages by developing alternative nutrient sources—from ancient organic amendments to modern industrial fertilizers and, increasingly, the recovery of nutrients from urban and agricultural waste streams (Antonkiewicz and Łabętowicz 2016).

Within this context, struvite (MgNH₄PO₄·6H₂O) precipitation has gained prominence as a low-energy and cost-effective technology for the simultaneous recovery of nitrogen and phosphorus (Siciliano et al. 2020). Beyond reducing nutrient discharges and eutrophication risks, struvite recovery aligns with circular economy principles by converting waste into value-added products (Lorick et al. 2020). The precipitation process follows the molar stoichiometry:

$${\mathrm{Mg}}^{2+}+{{\mathrm{NH}}_{4}}^{+}+{{\mathrm{PO}}_{4}}^{3-}+6{\mathrm{H}}_{2}\mathrm{O}\to {\mathrm{MgNH}}_{4}{\mathrm{PO}}_{4}\cdot 6{\mathrm{H}}_{2}\mathrm{O}$$

The efficiency of the process depends on operational parameters such as pH, molar ratios, ionic strength, temperature, and the presence of competing ions like Ca^2^⁺, which influence nucleation and crystal growth (Tai 2009; Korchef 2020). These physicochemical sensitivities shape product quality and agronomic performance.

Struvite is a slow-release fertilizer that supplies phosphorus, magnesium, and nitrogen, yielding consistent results across various cropping systems (Mancho et al. 2023). Large-scale applications, including full-scale recovery systems in wastewater treatment plants, highlight its technological feasibility and relatively low environmental footprint (Siciliano et al. 2020).

However, several challenges remain. Product characteristics, such as particle size, solubility, and crystalline stability, vary with production conditions and feedstock composition (Lorick et al. 2020). Structurally, phosphorus release is governed by the dissociation of PO₄ tetrahedra and Mg·6H₂O octahedra, whereas ammonium ions are less rigidly incorporated into the crystal lattice. However, occupying crystallographic sites is not integrated into the rigid polyhedral sub-network, leading to faster desorption (Yee et al. 2019; Bloem et al. 2024).

Concerns regarding the purity and safety of wastewater-derived struvite also persist. Studies have reported traces of heavy metals, pharmaceuticals, and microbial contaminants in wastewater-derived struvite, although, in some cases, the concentrations are lower than those in commercial fertilizers (Yee et al. 2019; Bloem et al. 2024). Still, occasional detections of cadmium, lead, and arsenic underscore the need for robust risk-assessment frameworks (Liu et al. 2021).

Phosphorus availability is further constrained by its fixation with iron and aluminum oxides in tropical soils, which limits the agronomic effectiveness of struvite (Hanyabui et al. 2020). Innovative approaches, such as co-application with acidifying agents, such as polysulfides, have demonstrated the potential to enhance phosphorus bioavailability under these conditions (Valle et al. 2022).

While phosphorus recovery from human urine and wastewater has been extensively studied, information on nutrient recovery from the effluents of dry toilet systems remains scarce. These systems present a distinct chemical profile due to the absence of water dilution and partial hydrolysis, which can influence struvite composition and its release behavior. Addressing this knowledge gap provides a novel perspective on decentralized sanitation and nutrient recycling research.

Thus, this study aims to advance the understanding of human urine struvite by characterizing its mineralogical and elemental composition, phosphorus speciation, concentrations of potentially toxic elements, and phosphorus release kinetics in water. This assessment provides critical insights into safety, performance, and potential of struvite as a regenerative fertilizer within decentralized sanitation systems.

## Material and methods

This study employed a structured experimental protocol divided into five integrated steps: (i) human urine collection, storage, and characterization, (ii) precipitation of human urine struvite, (iii) characterization of the precipitate, (iv) phosphorus speciation analysis, and (v) phosphorus release kinetics in aqueous media.

### Collection, storage, and characterization of source-separated human urine (HU)

The human urine was collected from dry sanitation systems, including urine-diverting toilet bowls, installed at a public university in Vitória, southeastern Brazil. The collected human urine was considered a waste stream for resource recovery.

A total of 450 L of HU effluent was stored in sealed 200-L polyethylene containers and maintained at ambient temperature (average 25ºC, INMET) for six months to enable natural biological sanitization, as recommended by Heinonentanski et al. (2007).

Preliminary analyses of the stored HU were performed in triplicate using standard analytical procedures: titrimetric methods for total Kjeldahl nitrogen (TKN) and ammonium (N-NH_4_^+^), colorimetric assays for total phosphorus and orthophosphate (PO_4_^3−^), and electrometric methods for pH. All procedures followed the guidelines of the Standard Methods for the Examination of Water and Wastewater (APHA 2012).

Additionally, density was measured gravimetrically according to the classical definition of density as the ratio of mass to volume (Higham adn Boyes 2003). A known volume of human urine (10 mL) was weighed on an analytical balance (± 0.1 mg) at 25 ºC, and results were expressed in g∙cm⁻^3^. The mean density recorded was 1.016 ± 0.002 g∙cm⁻^3^. Macro- and micronutrients (e.g., K, Ca, Mg, Na), and trace elements (Cd, Pb, Cr, Hg, Ni, Cu, Zn, Fe, Mn, Al) were measured by ICP-OES. All levels of heavy metals were below the maximum permissible limits established by the Brazilian legislation for mineral fertilizers (MAPA Ordinance No. 27/2006).

### Precipitation of struvite

Struvite precipitation was conducted using a six-vessel Jar Test apparatus with controlled agitation (up to 600 rpm). The stored HU was treated with magnesium oxide (MgO, 96% purity) at a concentration of 0.50 g/L, without pH adjustment to achieve a Mg:P molar ratio of 1.71:2.21, as proposed by (Ganrot et al. 2007).

**Fig. 1:**
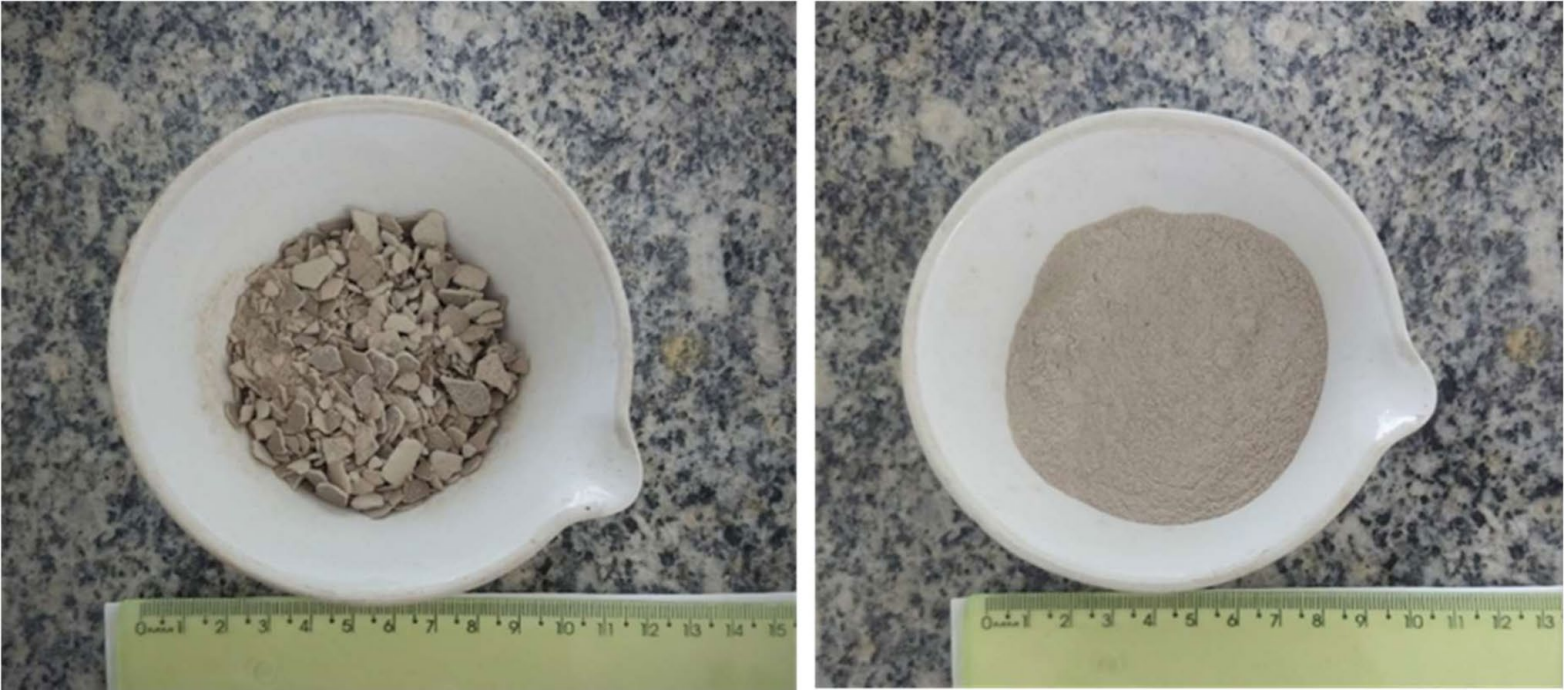
Precipitate after oven drying (left); Homogenized material after grinding and sieving (right)

The mixture was stirred at 100 rpm for ten minutes, followed by centrifugation for 30 min. After separation, the supernatant was discarded, and the precipitate was dried in a laboratory oven at 45 ºC for 120 h to preserve the crystalline purity of the particulate (Ali 2007). The dried material was then manually ground and sieved using a 35.5 µm mesh to obtain a fine and homogeneous particle size distribution (Fig. 1).

### Characterization of struvite

#### Mineralogical composition analysis

The mineralogical characterization of the human urine struvite precipitate was performed using X-ray diffraction (XRD), a technique widely recognized for its precision in identifying and quantifying crystalline phases in solid materials. About 0.375 g of the dried precipitate was additionally ground in a mortar to ensure sample homogenization and achieve the required particle size for analysis.

Diffractograms were obtained using a Shimadzu LabX XRD-6100 instrument, operating with CuKα radiation (λ = 1.5418 Å). Acquisition parameters included a 2θ scan range from 3º to 90º, a step size of 0.01°, a 0.5 mm slit, diverging lenses, and scintillation detection (Hall et al. 2020). These settings were selected to optimize peak resolution and ensure data reliability.

Mineral phases were identified by comparing diffraction peaks with crystallographic standards. The crystallographic references used for phase identification included orthorhombic struvite (MgNH₄PO₄∙6H₂O, PDF 15–762), hexagonal brucite (Mg(OH)₂, PDF 7–239), triclinic albite (NaAlSi₃O₈, PDF 1–739), monoclinic muscovite (KAl₂(Si₃Al)O₁₀(OH, F)₂, PDF 6–263), and tetragonal meionite (Ca₄Al₆Si₆O₂₄CO₃, PDF 2–405), with each phase identified by its corresponding crystal system, mineral name, and chemical formula to ensure accurate phase matching during X-ray diffraction analysis. Peak areas were calculated using Gaussian curve fitting, enabling the relative quantification of crystalline phases and the accurate determination of struvite content, as well as the identification of secondary phases.

#### ^31^P solid-state NMR spectroscopy

To complement the XRD analysis, solid-state nuclear magnetic resonance (NMR) spectroscopy was employed to investigate the chemical environment of phosphorus within the crystalline matrix. Spectra were acquired using a Varian/Agilent 400 MHz spectrometer operating at 9.4 T, with a resonance frequency of 161.8 MHz for the ^31^P nucleus. A 4 mm zirconia rotor was used to ensure thermal stability and high-speed rotation.

Cross-polarization (CP) was applied to enhance signal sensitivity, with a contact time of 1 ms and a recycle delay of 5 s. A total of 500 scans were acquired under magic-angle spinning (MAS) at 15 kHz, minimizing line broadening and improving spectral resolution. The spectral window was set to 50 kHz to encompass the expected chemical shifts (Hall et al. 2020). Data processing and interpretation were performed using OriginPro 8 software**.**

#### Elemental composition

Elemental analysis of precipitated struvite and reference fertilizers was performed according to USEPA Method 3051a (2007). Approximately 300 mg of each sample was digested in a CEM MD-4531 microwave oven using 10 mL of suprapure nitric acid (HNO₃), operated at 1600 W for five minutes at 170 °C. Samples were kept at this temperature for an additional 5 min, then gradually cooled to 20 °C over 60 min to prevent volatilization losses.

The digested solutions were diluted to 50 mL with ultrapure water. Three reagent blanks were prepared for quality control. Elemental concentrations were determined via inductively coupled plasma optical emission spectrometry (ICP-OES) using a PerkinElmer® Optima 7000DV spectrometer with an S10 autosampler (157 positions). Plasma and auxiliary gases were argon and nitrogen (99.999% purity), respectively. The ICP-OES operating conditions were as follows: the radio frequency generator operated at 40 MHz with a power setting of 1300 W. Gas flows were set to 15 L min⁻^1^ for the plasma gas, 0.8 L min⁻^1^ for the nebulizer gas, and 0.2 L min⁻^1^ for the auxiliary gas. The sample aspiration rate was 1.0 mL min⁻^1^, with a delay time of 45 s and a rinse time of 40 s.

Calibration curves were constructed using a certified multi-element standard solution (SpecSol), containing arsenic (As), aluminum (Al), barium (Ba), cadmium (Cd), calcium (Ca), cobalt (Co), copper (Cu), lead (Pb), chromium (Cr), iron (Fe), phosphorus (P), magnesium (Mg), manganese (Mn), nickel (Ni), mercury (Hg), selenium (Se), sodium (Na), potassium (K), and zinc (Zn). Five concentrations (10–50 mg.L⁻^1^) were used to ensure accurate quantification. For sulfur (S), a stock solution of sodium sulfate (Na₂SO₄) was diluted to match the same range.

Element selection was based on the typical composition of human urine (Karak and Bhattacharyya 2011), which is relevant for evaluating precipitation efficiency and fertilizer quality. Phosphorus content was referenced from the manufacturer's specifications for MAP. Spectral lines were selected according to Souza et al. (2014) and Hall et al. (2020) to minimize interference and optimize sensitivity. The wavelengths used for ICP-OES analysis were as follows: arsenic (As), 188.979 nm (ionic); aluminum (Al), 394.401 nm (atomic); barium (Ba), 233.527 nm (ionic); cadmium (Cd), 226.502 nm (ionic); calcium (Ca), 317.933 nm (ionic); cobalt (Co), 238.892 nm (ionic); copper (Cu), 324.752 nm (atomic); lead (Pb), 220.353 nm (ionic); chromium (Cr), 283.563 nm (ionic); sulfur (S), 181.975 nm (atomic); iron (Fe), 239.562 nm (ionic); phosphorus (P), 213.617 nm (atomic); magnesium (Mg), 285.213 nm (atomic); manganese (Mn), 259.372 nm (ionic); mercury (Hg), 194.168 nm (ionic); nickel (Ni), 231.604 nm (atomic); potassium (K), 766.975 nm (atomic); selenium (Se), 196.026 nm (atomic); sodium (Na), 588.995 nm (atomic); and zinc (Zn), 213.857 nm (ionic).

Equation 1 was used to calculate detection limits (LOD), as proposed by (Raya-Rodriguez and Albano 2015):

1 $$\mathrm{LOD}=\frac{Sa . 3}{IC}$$

In which, $Sa$ is the standard deviation obtained from ten consecutive readings of the calibration blank, and $IC$ is the slope of the calibration curve.

#### Total nitrogen analysis (Kjeldahl Method)

The Kjeldahl method was adapted to quantify total nitrogen in the precipitated struvite (APHA 2012). About 1000 mg of sample was digested with 20 mL of 0.203 mol∙L^−1^ sulfuric acid (H_2_SO_4_). Distillation was performed using a Marcone MA036 unit, with the addition of 30 ml of 40% sodium hydroxide (NaOH) and 30 ml of boric acid (H_3_BO_3_). Titration was conducted using standardized H_2_SO_4_ (1.0067 mol∙L^−1^). Analyses were performed in triplicate, with three blanks used as reagent controls. The nitrogen content in MAP and conventional urea (UC, 45% N) was obtained from the manufacturer's specifications.

### Phosphorus speciation analysis

Phosphorus speciation in the precipitate material was performed using the Measurement and Testing Protocol (MTP), which enables the identification and quantification of distinct phosphorus fractions and their associations with waste-derived nutrients (Ghanim et al. 2018). Although there are several methods for nutrient fractionation, the MTP was developed to standardize procedures and facilitate cross-study comparisons (Hedley et al. 1982). This protocol has not been applied to struvite; however, its use is supported by successful cases to analyze phosphorus in heat-treated poultry litter and untreated organic waste (Ghanim et al. 2018) as well as untreated waste (García et al. 2013; Ghanim et al. 2018).

The MTP protocol employs three extraction strategies to isolate five phosphorus fractions: total phosphorus (TP), inorganic phosphorus (IP), organic phosphorus (OP), apatite-bound phosphorus (PA), and non-apatite phosphorus (PNA). After extraction, phosphorus concentrations and associated elements were quantified using a PerkinElmer Optima 7000DV ICP-OES spectrometer.

#### Total phosphorus (TP) extraction

TP was determined via dry combustion. Approximately 200 mg of the precipitated struvite was placed in porcelain crucibles and incinerated in a muffle furnace at 450ºC for three hours. After cooling, 20 mL of 3.5 mol∙L^−1^ HCl was added to each sample, followed by shaking for 15 min. The resulting suspensions were centrifuged at 2000 g for 15 min, and the supernatants were filtered for TP quantification.

#### Inorganic (IP) and organic phosphorus (OP) extraction

Sequential extraction was used to separate IP and OP fractions based on their differential solubility in HCl. For IP, 200 mg of sample was mixed with 20 mL of 1.0 mol∙L^−1^ HCl, shaken for 16 h, centrifuged at 2000 g for 15 min, and filtered. The supernatant was used to quantify IP.

To extract OP, the residue from the previous step was washed twice with ultrapure water, shaken for five minutes, and centrifuged again. The supernatant was discarded, and the remaining solid was calcined at 450 ºC for three hours. After cooling, 20 mL of 1.0 mol∙L^−1^ HCl was added, followed by shaking for 16 h and centrifugation. The filtered supernatant was used to determine OP.

#### Apatite (PA) and non-apatite (PNA) phosphorus extraction

PA and PNA fractions were extracted using alkaline and acid media, targeting phosphorus bound to calcium salts and amorphous iron/aluminum complexes. For PNA, 200 mg of sample was treated with 20 mL of 1.0 mol∙L^−1^ NaOH, shaken for 16 h, and centrifuged at 2000 g for 15 min. A 10 mL aliquot of the supernatant was mixed with 4 mL of 3.5 mol∙L^−1^ HCl, shaken for 20 s, and left to rest for 16 h. After centrifugation and filtration, the supernatant was used to quantify PNA.

The residue from the previous step was washed twice with ultrapure water and treated with 20 mL of 1.0 mol∙L^−1^ HCl to extract PA. The filtered extract was used to determine PA after shaking for 16 h and centrifugation.

### Phosphorus release in water

Phosphorus solubilization kinetics in aqueous media were evaluated to simulate nutrient availability under soil conditions typical of Brazilian agroecosystems. The procedure was adapted from Zhang et al. (2015) to assess the solubilization behavior of struvite-derived phosphorus across a range of pH values.

Approximately 5000 mg of struvite was mixed with 1000 mL of distilled water in a reaction vessel. The pH was adjusted using sulfuric acid (H_2_SO_4_) to reflect the common soil pH levels (4.0 to 7.5). Five parallel tests were conducted: one at natural water pH (~ 7.0), three at adjusted pH levels (4.0, 5.0, and 6.0), and one blank to control reagent contamination.

The mixtures were stirred at 120 rpm using a Nova Ética Jar Test apparatus for up to 240 h. Aliquots of 25mL were collected at the following time intervals: 0.25, 0.50, 1.0, 6.0, 12, 24, 48, 72, 120, and 240 h. Temperature and pH were recorded at each sampling point, and mass losses during agitation were considered negligible.

Phosphorus concentrations were determined by colorimetry, following the method of Murphy and Riley (1962). The release kinetics were modeled using the Elovich equation (Eq. 2), which describes cumulative phosphorus release over time:

2 $${K}_{t}=a+b\bullet \mathrm{ln}\left(t\right)$$

In which, ${K}_{t}$ is the cumulative phosphorus released at time $t$, $a$ is the initial release rate, and $b$ is the desorption resistance constant.

The model’s fit was evaluated using the coefficient of determination (R^2^), which provided insight into release dynamics under varying pH conditions.

## Results and discussion

### Physicochemical dynamics of human urine effluent during storage

The initial characterization of three human urine samples revealed the following mean values: pH of 5.9 ± 0.2, density of 1.016 ± 0.002 g∙cm^−3^, total Kjeldahl nitrogen (TKN) 6.946 ± 1,371 mg∙L^−1^, total phosphorus 874 ± 53 mg∙L^−1^, potassium 1,535 ± 121 mg∙L^−1^, calcium 102 ± 8 mg/L, and magnesium 53 ± 5 mg∙L^−1^. Heavy metal concentrations were negligible, with Ni, As, and Pb below 0.005 mg∙L^−1^, and Hg and Cd below 0.001 mg∙L^−1^.

After six months of ambient storage, significant changes were observed: the pH increased markedly to 10.0 ± 0.2, TKN decreased to 5,557 ± 1,047 mg∙L^−1^, total phosphorus to 744 ± 45 mg∙L^−1^, while potassium increased slightly to 1,621 ± 32 mg∙L^−1^. Calcium and magnesium concentrations dropped to 61 ± 5 mg∙L^−1^ and 23 ± 2 mg∙L^−1^, respectively. These trends are consistent with previous studies (Udert et al. 2003), reinforcing the reliability of the experimental protocol.

The pH elevation and nutrient reduction are indicative of urea hydrolysis (ammonification), a process that promotes spontaneous precipitation of minerals such as struvite (MgNH₄PO₄·6H₂O) and calcium phosphate (Ca₃(PO₄)₂) (Tilley et al. 2008). The shift from acidic to alkaline conditions (pH > 9.0) facilitates chemical reactions that remove nitrogen and phosphorus from solution (Jagtap and Boyer 2018).

Zancheta (2012) reported similar behavior, with total phosphorus decreasing from 700 to 500 mg∙L^−1^ and orthophosphate from 580 to 470 mg∙L^−1^ after 37 days of storage. Cancian et al. (2025) noted that up to 50% of nitrogen may be lost under elevated temperature and pH conditions. Udert et al. (2003) further observed that urea hydrolysis can occur within minutes, resulting in the volatilization of ammoniacal nitrogen.

Based on the concentration differences before and after storage (ΔP = 130 mg∙L^−1^, ΔCa = 41 mg∙L^−1^, and ΔMg = 30 mg∙L^−1^), the mass of spontaneously precipitated solids was estimated using stoichiometric calculations. The precipitation of 30 mg·L⁻^1^ Mg^2^⁺ corresponds to 1.23 mmol·L⁻^1^, which translates into a potential formation of 301.85 mg·L⁻^1^ struvite (MgNH₄PO₄·6H₂O).

Similarly, the precipitation of 41 mg·L⁻^1^ Ca^2^⁺ (equivalent to 1.02 mmol·L⁻^1^) would theoretically yield 0.34 mmol·L⁻^1^ of calcium phosphate (Ca₃(PO₄)₂), corresponding to 105.46 mg·L⁻^1^. Total estimated mass of suspended solids (TSS) was 0.41 g·L⁻^1^ (struvite and calcium phosphate), confirming substantial nutrient removal via spontaneous precipitation.

### Experimental yield of human urine struvite and nutrient recovery compared to theoretical potential

The addition of MgO to 450 L of stored human urine effluent resulted in 940 g of precipitate, corresponding to a mean yield of 2.09 g·L⁻^1^. Elemental analysis of the dried material revealed 10.2% phosphorus and 3.43% nitrogen, equivalent to 96 g of P and 32 g of N recovered as struvite (nutrient removal efficiencies of 1.3% for N and 28.7% for P).

Table 1 predicts the theoretical maximum values, which were substantially lower (432 g of P and 2.138 g of N, respectively), most likely due to ammonia volatilization and operational limitations, including reactor design, pH control (optimal range: 8–10), and molar ratios of Mg:P (Ganrot et al. 2007).

**Table 1:** Theoretical N and P recovery potential from stored human urine

Parameter	Quantity	Unit
Human urine volume	450	Liters
Unit production	1.5	L/hab.d
Equivalent population	300	Hab
Unit nitrogen	8.91	g N/hab.d
Nitrogen loss in six months	20	%
Nitrogen excreted by the population	2.138	g N
Unit phosphorus	1.44	gP/hab.d
Phosphorus	432	g P

The experimental yields obtained in this study exceeded those reported by Etter et al. (2011), who recovered 1.56 g∙L^−1^ of struvite at pH 9.0 and a Mg:P molar ratio of 1.5. Similarly, Antonini et al. (2011) achieved up to 2.24 g∙L^−1^, with nutrient removal efficiencies of 94% for N and 98% for P under pH conditions ranging from 9.1 to 9.6.

These findings highlight the importance of carefully regulating operational parameters, particularly pH and Mg:P molar ratios, to optimize struvite precipitation and enhance nutrient recovery in decentralized sanitation systems.

### Integrated analysis of struvite precipitates

XRD enabled the identification of crystalline phases, while ^31^P NMR provided insights into the chemical environment of phosphorus, distinguishing between amorphous and crystalline forms. These complementary techniques are essential for evaluating the structural integrity and agronomic potential of the recovered fertilizer.

**Fig. 2:**
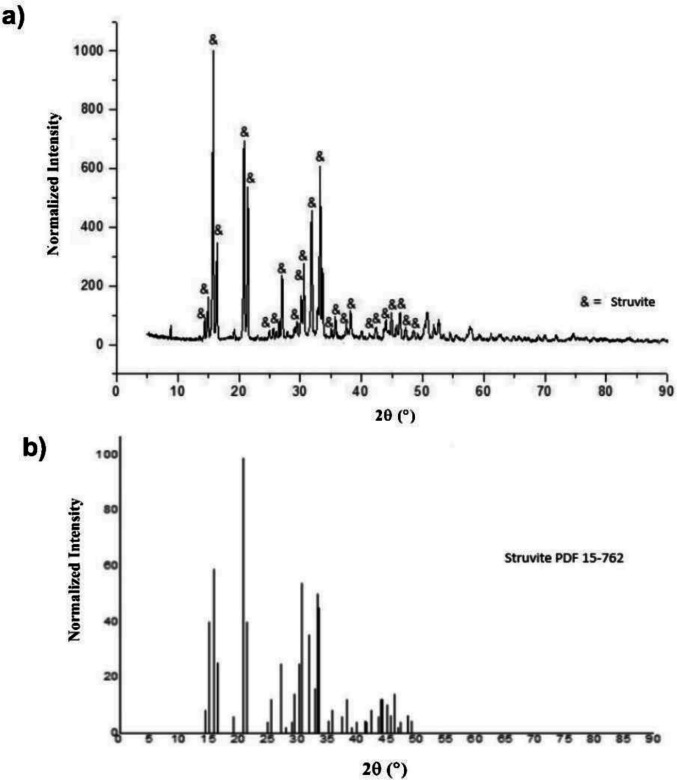
**a**) XRD diffractogram of the precipitated material; **b**) Reference XRD diffractogram for struvite. Source: LMC crystallographic database (2021)

Figure 2a shows the XRD diffractogram of the precipitated material, and Fig. 2b shows the reference pattern for struvite. The high similarity between the observed peaks confirms that struvite (MgNH₄PO₄·6H₂O) is the predominant crystalline phase.

**Fig. 3:**
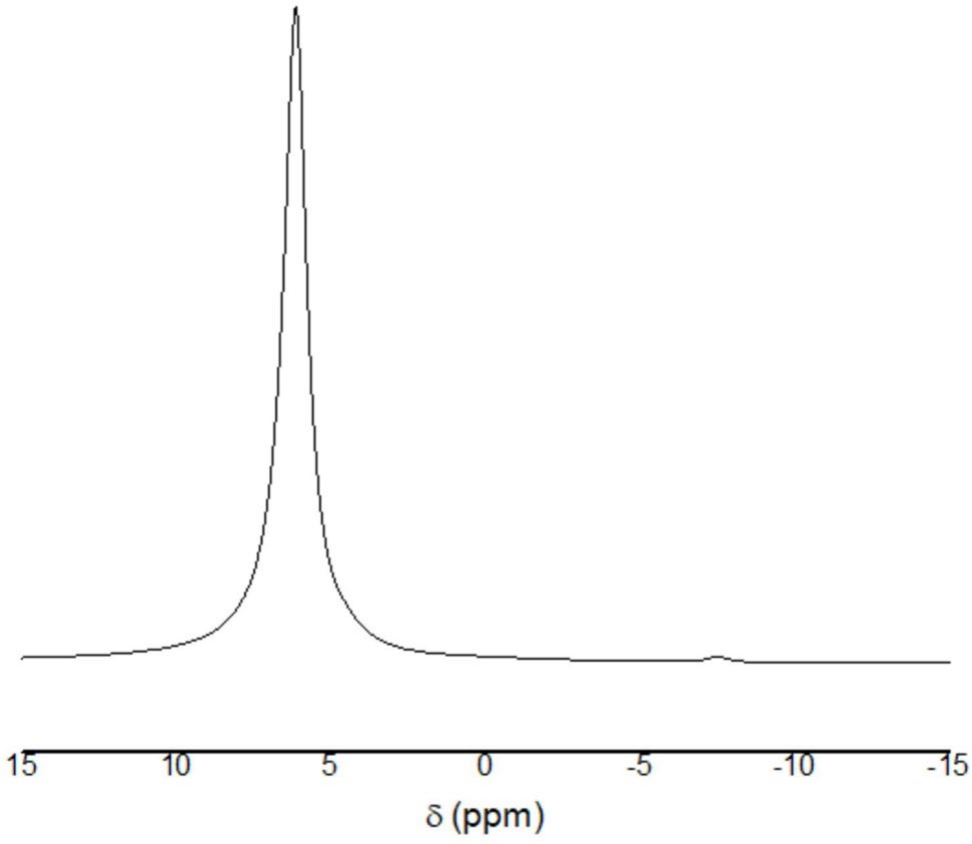
Solid-state ^31P NMR^ spectrum of the precipitated material

The ^31P NMR^ spectrum showed a dominant signal between 6.3 and 6.4 ppm, consistent with the struvite spectral signature (Bak et al. 2000) and corroborating the XRD results. A secondary peak at −7.2 ppm (Fig. 3) was attributed to newberyite (MgHPO₄·3H₂O), an amorphous phase commonly found in urine-derived precipitates (Hunger et al. 2004) and not detectable by XRD. The simultaneous identification of both phases underscores the importance of integrated analytical approaches for precise mineralogical characterization.

**Fig. 4:**
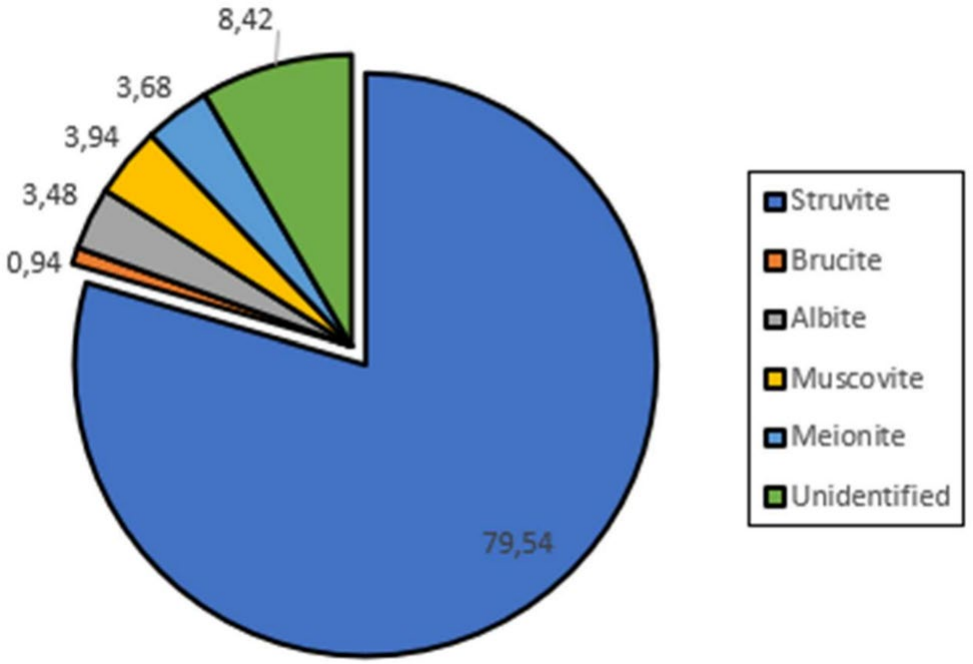
Relative abundance of mineral phases in the precipitated material

Quantitative analysis of crystallographic data estimated the mineralogical composition to be 79.5% struvite (Fig. 4). Minor phases included muscovite (3.94%), meionite (3.68%), albite (3.48%), and brucite (0.94%), while 8.42% of the material remained unidentified by XRD. Among these, only muscovite contains agronomically relevant elements (potassium), while the others do not contribute nitrogen or phosphorus.

These findings align with previous studies. Antonini et al. (2012) reported struvite as the dominant phase in urine-derived precipitates, with proportions ranging from 74 to 100%. Other researchers have identified secondary phases such as dittmarite ((NH₄)Mg(PO₄) · H₂O), nesquehonite (Mg(HCO₃)(OH) · 2H₂O), and silvine (KCl) (Bak et al. 2000). Although newberyte lacks ammonium, which reduces its fertilizing potential, Hall et al. (2020) suggest that its presence at low levels does not significantly impair phosphorus release in aqueous media.

The elemental composition of the precipitated struvite, determined by ICP-OES as described in Section "Elemental composition", showed mean macronutrient levels of 10.20% P, 6.99% Mg, 1.89% Ca, 1.09% K, 0.12% S, and 3.43% N. Although pure struvite theoretically contains 12.66% P, 9.9% Mg, and 5.7% N, the values obtained are consistent with those of struvite recovered from other waste matrices, such as sewage sludge and organic residues (Cabeza et al. 2011; Talboys et al. 2016; Degryse et al. 2017). The lower nitrogen content is attributed to volatilization during storage and processing, as also reported by Antonini et al. (2011).

Heavy metal concentrations in the human urine struvite were compared against Brazilian regulatory limits for mineral fertilizers containing nitrogen, potassium, secondary macronutrients, or up to 5% phosphorus pentoxide (P₂O₅), as established by Normative Instruction SDA N^o^. 27/2006, amended by IN SDA No. 7/2016 (Table 2).

**Table 2:** Heavy metal concentrations and regulatory limits

Element	Human urine struvite (mg∙kg^−1^)	Permitted limit (mg∙kg^−1^)
Al	296.89 ± 19.31	-
As	2.01 ± 0.12	10
Ba	9.33 ± 0.42	-
Cd	0.08 ± 0.01	20
Cr	6.14 ± 0.06	200
Cu	110.94 ± 6.45	250
Fe	169.51 ± 16.20	-
Hg	< LD	0.2
Mn	76.40 ± 19.89	-
Na	9774.9 ± 104.9	-
Nb	< LD	-
Ni	0.94 ± 0.08	50
Pb	5.89 ± 0.07	100
Se	0.27 ± 0.04	2.0
Ti	4.90 ± 0.15	-
Zn	175.43 ± 30.93	2.500

*LD: Limit of Detection Note: Concentrations of trace metals and metalloids in human urine–derived struvite compared with Brazilian regulatory limits for mineral fertilizers (IN SDA No. 27/2006, amended by IN SDA No. 7/2016)

The low concentrations of heavy metals are attributed to the origin of the raw material – Human urine, which naturally contains minimal levels of contaminants. Consequently, the incorporation of metal into struvite crystals is limited. In contrast, struvite recovered from anaerobic sewage sludge or livestock effluents often shows elevated metal content. Uysal et al. (2010) reported Fe levels of up to 71%, As levels of up to 60%, and Hg levels of up to 49% in sludge-derived struvite. Liu et al. (2011) found 43% Zn and 37% Cu in struvite from pig farm wastewater.

Muys et al. (2021) confirmed that metal levels remained within legal limits by analyzing 24 struvite samples from European facilities. Reza et al. (2019) observed similar results in pig farm effluent-derived struvite, while Shen et al. (2016) reported elevated Cu and Zn concentrations in comparable materials. These findings reinforce the importance of comprehensive chemical and mineralogical characterization to ensure the agronomic safety and environmental sustainability of struvite-based fertilizers. Compared with struvite obtained from conventional urine or sludge digestates, the material recovered in this study showed compositional differences linked to the distinctive chemistry of dry toilet effluents, confirming the uniqueness of this matrix.

### Phosphorus speciation in human urine effluent-derived struvite

The agronomic effectiveness of phosphorus fertilizers is closely linked to phosphorus speciation and soil environmental conditions. In this study, the total phosphorus (TP) concentration in the struvite-based material was 71.6 mg∙g^−1^, confirming its potential as a concentrated source of this essential macronutrient.

Most phosphorus was found in the inorganic fraction (IP = 70.0 mg∙g^−1^), while the organic fraction (OP = 12.3 mg∙g^−1^) represented a smaller proportion. Inorganic phosphorus, particularly in the form of ammonium magnesium phosphate (struvite), is readily available to plants (Doyle and Parsons 2002). In contrast, organic phosphorus requires microbial mineralization in soil to become bioavailable, resulting in a gradual, sustained release (Le Corre et al. 2009).

The material also contained non-apatite (PNA = 27.6 mg∙g^−1^) and apatite-bound phosphorus (PA = 12.6 mg∙g^−1^). PNA is typically associated with amorphous or organic complexes and tends to be more soluble and accessible to plants (Celi et al. 2023). PA, found in crystalline minerals, has lower solubility, and its availability is influenced by soil pH, microbial activity, and the presence of solubilizing agents (He and Zhu 1998). Agronomic strategies such as the use of phosphorus-solubilizing microorganisms and acidifying amendments can enhance PA utilization (Khan et al. 2024).

The organic phosphorus content in this study was lower than that reported for matrices rich in organic matter, such as sewage sludge and poultry litter (Table 3). However, González Medeiros et al. (2005) emphasize that organic phosphorus levels do not necessarily correlate with total organic matter, supporting the findings of Ghanim et al. (2018).

**Table 3:** Phosphorus fractionation in struvite-based fertilizer (mean ± SD, n = 3) and comparative data from the literature

Sample	IP (mg∙g^−1^)	OP (mg∙g^−1^)	PNA (mg∙g^−1^)	PA (mg∙g^−1^)	TP (mg∙g^−1^)	Reference
Human urine effluent-derived struvite	70.0 ± 3.50	12.3 ± 0.61	27.6 ± 1.65	12.6 ± 0.53	71.6 ± 2.90	This research
Sewage sludge-derived struvite	27.4 ± 1.04	0.802 ± 0.05	8.8 ± 0.45	14.1 ± 0.96	31.2 ± 1.99	(García-Albacete et al. 2012)
Poultry litter-derived struvite	8.50 ± 0.42	0.89 ± 0.01	3.3 ± 0.10	3.29 ± 0.20	14.6 ± 0.47	Ghanim et al. (2018)
Poultry litter-derived pyrolytic hydrocarbons (225 °C, 5 min)	14.6 ± 0.02	0.11 ± 0.01	2.9 ± 0.06	8.3 ± 0.12	15.8 ± 0.14	Ghanim et al. (2018)
Poultry litter-derived pyrolytic hydrocarbons (200 °C, 5 min)	26.8 ± 0.27	0.15 ± 0.00	16.9 ± 0.11	3.2 ± 0.04	28.2 ± 0.10	Ghanim et al. (2018)
Poultry litter-derived pyrolytic hydrocarbons (250 °C, 5 min)	36.7 ± 1.08	0.19 ± 0.01	25.0 ± 0.16	25.0 ± 0.16	37.3 ± 0.39	Ghanim et al. (2018)

IP = inorganic phosphorus, OP = organic phosphorus, PNA = non-apatite phosphorus, PA = apatite phosphorus, TP = total phosphorus

The mineral composition of the fertilizer is strongly influenced by the chemical profile of human urine and the use of MgO as a precipitating reagent. Understanding the elemental distribution across phosphorus fractions enables the estimation of nutrient stability and solubility, which are critical aspects for soil release dynamics (Ghanim et al. 2018) (Table 4).

**Table 4:** Elemental concentrations (mg.g^−1^) in phosphorus fractions (MTP protocol)

Element	IP	OP	PNA	PA	TP
Ca	15.82	14.20	0.02	16.39	14.41
K	10.10	3.46	24.71	3.91	11.04
Na	8.01	3.74	1.03	56.86	8.83
Mg	50.84	12.36	0.02	68.83	51.91
S	0.84	0.20	0.46	0.01	1.29
Fe	0.09	0.96	0.01	0.11	0.15
Mn	0.04	0.02	0.01	0.06	0.05
Al	0.15	0.11	0.04	0.15	0.16
Cu	0.08	0.03	0.04	0.01	0.08
Zn	0.26	0.14	0.06	0.26	0.28

The data reveal that K is predominantly concentrated in the PNA fraction (24.71 mg · g^−1^), consistent with findings by Ghanim et al. (2018) for poultry litter-derived hydrocarbons. In contrast, Ca, Na, and Mg were more abundant in the PA fraction, which may explain their reduced solubility and slower release rates. Other elements, such as S, Fe, Mn, Al, Cu, and Zn, were present at modest concentrations across all fractions, suggesting minimal interference with phosphorus mobility and bioavailability.

### Phosphorus release kinetics in water

**Fig. 5:**
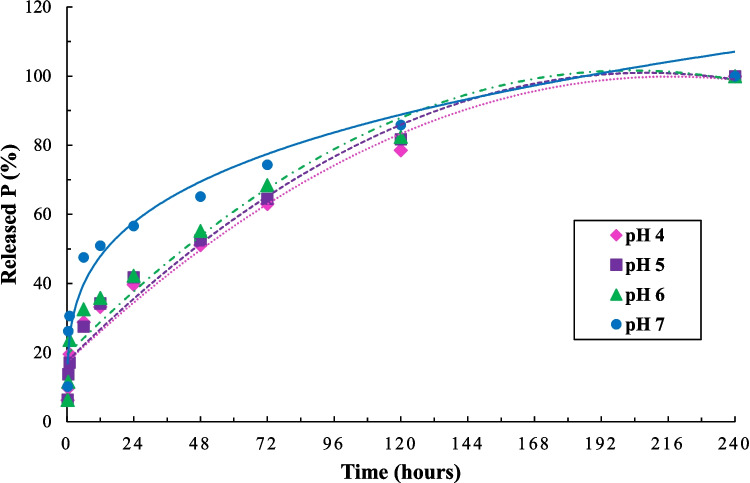
Phosphorus release profile over time under different pH conditions

The pH of the aqueous medium strongly influenced the release dynamics of phosphorus from the human urine struvite. Across all tested pH levels, a gradual increase in soluble phosphorus concentration over time was observed, with the system at neutral pH (7.0) showing the highest release (Fig. 5). Although acid conditions typically enhance solubilization through proton-driven dissolution reactions, the superior performance at pH 7 suggests that struvite exhibits optimal ionic stability in this range, enabling controlled and sustained desorption (Kim et al. 2017).

Kinetic modeling of phosphorus release revealed an excellent fit to the Elovich model (Table 5), which is commonly applied to describe desorption on heterogeneous surfaces (Jalali and Ahmadi Mohammad Zinli 2011). The coefficients of determination (R^2^) ranged from 0.88 to 0.97, indicating strong agreement between experimental data and model predictions. The highest $a$ value was recorded at pH 7, confirming a more rapid initial release. Lower $a$ values at pH 4, 5, and 6 suggest slower release kinetics, likely governed by diffusion-limited processes.

**Table 5:** Adjusted Elovich equations (${K}_{t}=a+b\bullet \mathrm{ln}\left(t\right)$) for each pH condition

pH	a	b	R^2^
4	2.835	19.360	0.88
5	3.5170	25.653	0.88
6	3.3812	25.601	0.97
7	6.3781	35.901	0.96

After 240 h of agitation, the cumulative phosphorus release at pH 7 reached 62.5 g∙kg^−1^, corresponding to 61% of the total phosphorus content in the fertilizer (102 g∙kg^−1^). At pH 4, 5, and 6, the respective release values were 45 (44%), 55 (54%), and 53 (52%) g∙kg^−1^. The release behavior observed supports the classification of struvite as a slow-release phosphorus source, particularly effective in tropical soils with high phosphorus fixation. This gradual release aligns with crop nutrient demands, enhancing uptake efficiency and minimizing losses due to adsorption onto soil colloids (Teixeira et al. 2016).

Unlike conventional phosphate fertilizers, which show rapid solubilization and limited residual effect, struvite offers a strategic alternative for long-cycle crops and low-input systems, contributing to improved nutrient management and sustainability. Its performance under varying pH conditions further supports its suitability for diverse soil types, especially in regions with acid or highly weathered soils, where phosphorus availability is typically constrained.

## Conclusion

This study demonstrated the technical feasibility and environmental relevance of producing a struvite-based fertilizer from human urine via a simplified process leveraging natural microbial activity. Struvite was confirmed as the predominant crystalline phase (79.54%) via comprehensive structural and elemental characterization, with minor presence of amorphous phase newberyite, as revealed by complementary XRD and solid-state ^31^P NMR analyses, the latter being crucial for understanding the nuanced mineralogical contributions to its slow-release behavior. The material showed high concentrations of phosphorus (10.2%), magnesium (6.99%), and nitrogen (3.43%), while maintaining the levels of heavy metals well below regulatory thresholds, unequivocally ensuring its agronomic safety. Phosphorus speciation via the MTP protocol revealed a predominance of inorganic forms (e.g., struvite and newberyite-associated phosphorus), which are readily bioavailable. The kinetic release tests confirmed a slow, sustained phosphorus desorption profile, especially at neutral pH, with an excellent fit to the Elovich model. This behavior is desirable for enhancing nutrient use efficiency and minimizing environmental losses via leaching or fixation. Collectively, these findings position HU-derived struvite as a safe, efficient, and regenerative fertilizer, aligned with the principles of circular economy, resource recovery, and sustainable sanitation. Its gradual nutrient release is particularly advantageous in tropical soils, where phosphorus fixation is a limiting factor for crop productivity. As a limitation, the study focused on physicochemical and release assessments in aqueous media, without the direct evaluation of agronomic performance under field conditions. Future research should include field trials across diverse crops and soil types, and economic analyses of the struvite recovery process at full scale to validate its practical applicability and cost-effectiveness.

## Data Availability

The datasets generated during and/or analyzed during the current study are available from the corresponding author on reasonable request.
